# Regulating Blood Clot Fibrin Films to Manipulate Biomaterial-Mediated Foreign Body Responses

**DOI:** 10.34133/research.0225

**Published:** 2023-09-15

**Authors:** Yang Zou, Zhengjie Shan, Zongpu Han, Jieting Yang, Yixiong Lin, Zhuohong Gong, Lv Xie, Jieyun Xu, Runlong Xie, Zhuofan Chen, Zetao Chen

**Affiliations:** Hospital of Stomatology, Guanghua School of Stomatology, Sun Yat-sen University and Guangdong Research Center for Dental and Cranial Rehabilitation and Material Engineering, Guangzhou 510055, China.

## Abstract

The clinical efficacy of implanted biomaterials is often compromised by host immune recognition and subsequent foreign body responses (FBRs). During the implantation, biomaterials inevitably come into direct contact with the blood, absorbing blood protein and forming blood clot. Many studies have been carried out to regulate protein adsorption, thus manipulating FBR. However, the role of clot surface fibrin films formed by clotting shrinkage in host reactions and FBR is often ignored. Because of the principle of fibrin film formation being relevant to fibrinogen or clotting factor absorption, it is feasible to manipulate the fibrin film formation via tuning the absorption of fibrinogen and clotting factor. As biological hydroxyapatite reserved bone architecture and microporous structure, the smaller particle size may expose more microporous structures and adsorb more fibrinogen or clotting factor. Therefore, we set up 3 sizes (small, <0.2 mm; medium, 1 to 2 mm; large, 3 to 4 mm) of biological hydroxyapatite (porcine bone-derived hydroxyapatite) with different microporous structures to investigate the absorption of blood protein, the formation of clot surface fibrin films, and the subsequent FBR. We found that small group adsorbed more clotting factors because of more microporous structures and formed the thinnest and sparsest fibrin films. These thinnest and sparsest fibrin films increased inflammation and profibrosis of macrophages through a potential signaling pathway of cell adhesion–cytoskeleton–autophagy, leading to the stronger FBR. Large group adsorbed lesser clotting factors, forming the thickest and densest fibrin films, easing inflammation and profibrosis of macrophages, and finally mitigating FBR. Thus, this study deepens the understanding of the role of fibrin films in host recognition and FBR and demonstrates the feasibility of a strategy to regulate FBR by modulating fibrin films via tuning the absorption of blood proteins.

## Introduction

Biomaterials have been extensively applied in various biomedical fields [1]. As a foreign body, host immune system can recognize it and cause foreign body response (FBR), thus wrapping, degrading, or expelling the implants [2,3]. Understanding how the biomaterials mediate FBR has been important for improving the materials’ biocompatibility and long-term functioning. After implantation, biomaterials quickly interact with host blood, and a surface absorbed protein layer is then formed [4,5]. The adsorbed proteins can be recognized by immune system, recruiting macrophages to the surface of the implanted material and coordinating the fibrosis response of fibroblasts, leading to fiber encapsulation, which were generally accepted processes about FBR [6–10]. Therefore, many studies have been carried out to regulate protein adsorption, thus manipulating FBR [11–14]**.**

However, during the formation of blood clotting, fibrinogen is converted into a network of fibrin polymers, and then clots contract [15–17]. Contracted blood clots generate a remarkable structure, with dense fibrin films on the surface of the contracted clots [18]. These fibrin films may separate the biomaterials from surrounding tissue and keep the implanted material–blood clot mixture into a relatively isolated lesion environment [19]. The isolated environment may avoid microdamage caused by direct friction between the surrounding tissue and implants, minimizing the direct effect of biomaterial surface characteristics on cell responses, demonstrating a bioprotective effect [19–28]. It is reasonable to speculate that in addition to the surface protein layer, the fibrin film should also elicit important effects on regulating this foreign body and host response progress. Controlling fibrin film to control FBR would be of great interest.

The formation of clots fibrin including fibrin films on the surface of clots requires fibrinogen and clotting factors [18,27,29]. Clotting factor is the key catalyst for the conversion of soluble fibrinogen into insoluble fibrin, which can assemble fibrinogen into clots fibrin including the surface fibrin films [18,27,29]. As more fibrinogen or clotting factor absorbed into the biomaterials, ingredients including fibrinogen or clotting factor become less in the remaining blood, which may lead to the changes of clots fibrin including the surface fibrin films [18,27,29]. Therefore, we can manipulate the fibrin film formation via tuning the absorption of fibrinogen and clotting factor. Biogenic hydroxyapatite is a commonly used bone regeneration and repair material in orthopedics and dentistry, and the market demand is great. It has been well documented to be capable of absorbing fibrinogen and clotting factor, due to its reserving bone architecture and microporous structure during preparation [30–36]. As the biomaterial is porous, the smaller particle size may expose more microporous structures and adsorb more proteins [37]. Therefore, tuning the porcine bone-derived hydroxyapatite (PHA) particle size can manipulate the microporous structure, thus regulating the absorption of blood protein and the subsequent formation of clot surface fibrin films.

**Fig. 1.**
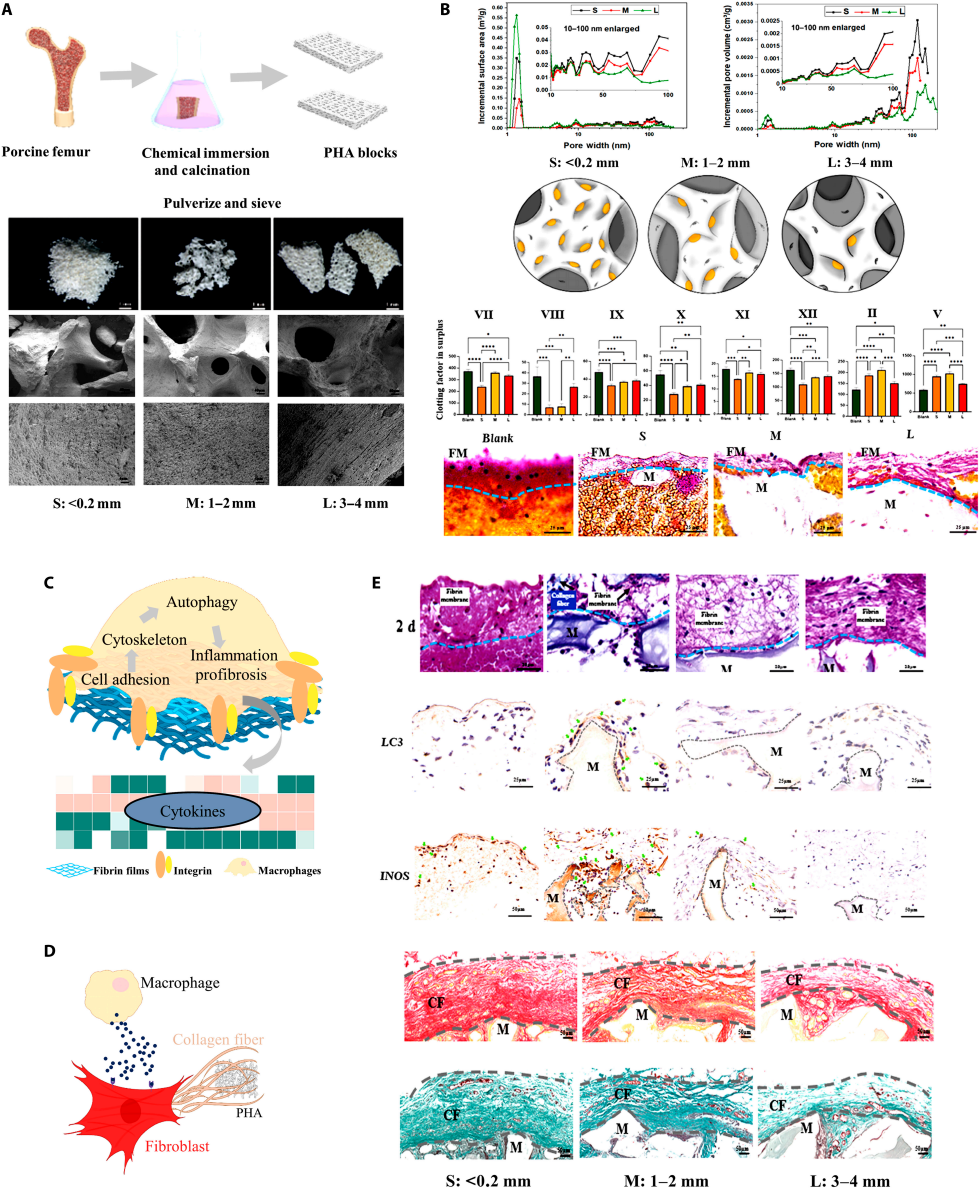
Experimental flow of the study. (A) The preparation of PHA particles with 3 different sizes. (B) Different numbers of microporous structures lead to different numbers of clotting factor absorption and then different absorptions leading to different clotting factors in the remaining blood, leading to different clot fibrins including surface fibrin film formation. (C) The thickness and density of fibrin films affect macrophages through a potential pathway of cell adhesion–cytoskeleton–autophagy. (D) Activated macrophages by fibrin films regulate the collagen secretion and collagen matrix of fibroblasts. (E) Thinner and sparser fibrin films induce obvious FBR, while thicker and denser fibrin films induce low FBR in vivo. *P < 0.05; **P < 0.01; ***P < 0.001; ****P < 0.0001.

We first set up 3 sizes of [small (S), <0.2 mm; medium (M), 1 to 2 mm; large (L), 3 to 4 mm] PHA to show the effect of microporous structure with different degrees of exposure on fibrinogen or clotting factor absorption and the regulation of fibrinogen or clotting factor absorption on clot surface fibrin film formation. Then, the effects of manipulated fibrin films on FBR and its underlying mechanisms were unveiled (Fig. 1). This study deepens the understanding of host recognition and FBR in implanted biomaterials and proposes an effective strategy to manipulate this clots surface fibrin films to control FBR, which may pave a new way for the development of advanced FBR-tunable biomaterials.

## Results and Discussion

### Preparing 3 sizes of PHA particles with microporous structure

PHA particles were prepared through chemical immersion and calcination according to our previous studies [34], and the intended particle sizes were controlled by sieves with 3 pore sizes (Fig. S1A). X-ray diffraction pattern results of the PHA showed that most peaks were matched with the hydroxyapatite (JCPDS 74-0565), and the Fourier transform infrared spectra results exhibited most representative bands of hydroxyapatite, suggesting the successful preparation of PHA (Fig. S1B), which were further characterized by scanning electron microscopy (SEM) showing their biomimic porous structure and well-controlled particle size. Among them, S group has the most PHA particle fracture surface, and every fracture surface has microporous structure, so S group has the most exposed microporous structure (Fig. S1C).

### The effects of PHA particle size on the clot surface fibrin film formation

The amount of protein absorption is highly dependent on the matching rate between the pore size and the protein size [38–39]. Pore size and distribution affect protein adsorption through regulating the surface area and pore volume [38–41]. The size of most clotting factors is between 10 and 100 nm [42], which means that the more microporous structures with a size of 10 to 100 nm, the more clotting factors will be absorbed. The more clotting factor absorbed by the microporous structures, the less clotting factor in the remaining blood, leading to the deficiency of fibrin formation and thinner and sparser surface fibrin film formation, and vice versa.

**Fig. 2.**
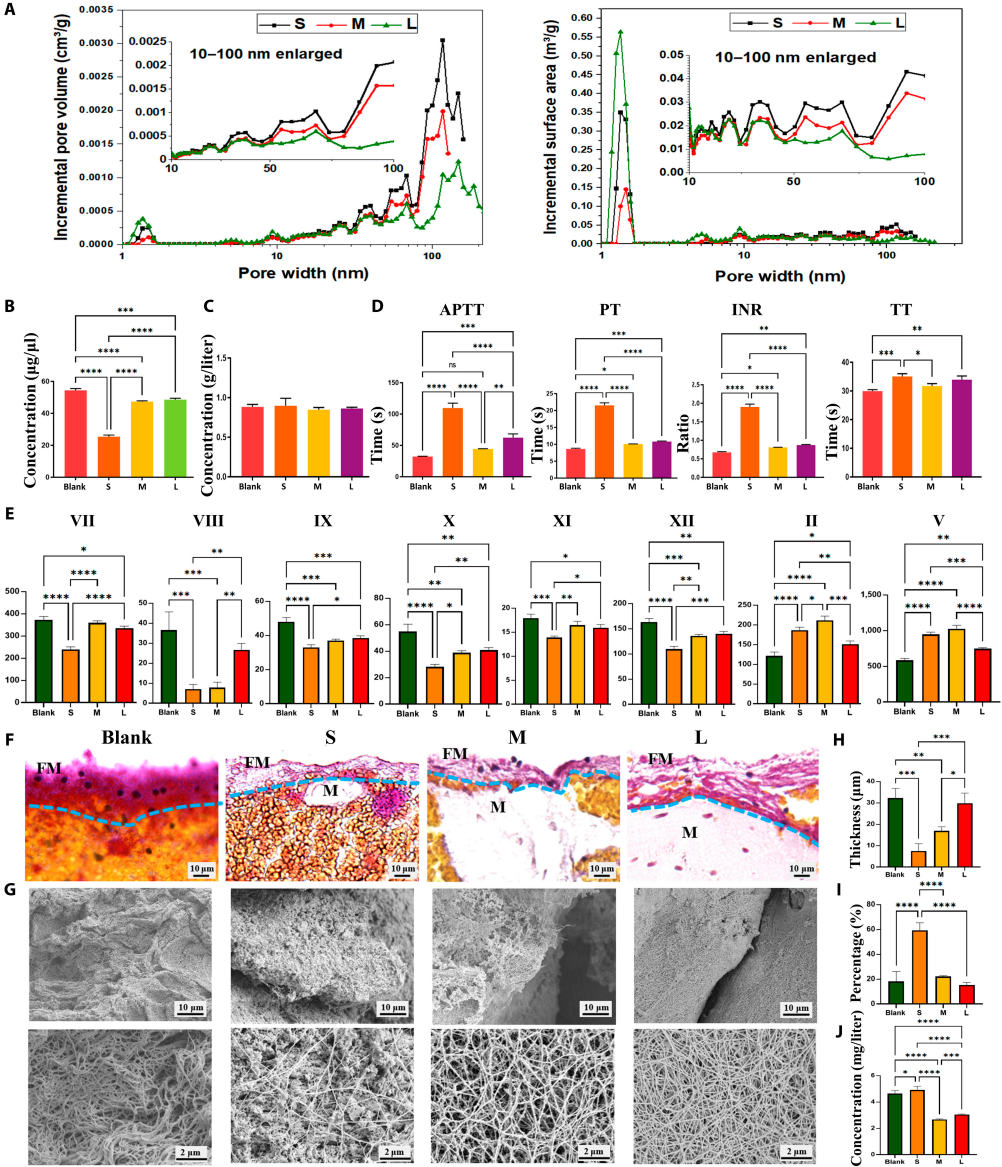
The effects of 3 sizes of PHA on fibrin films. (A) Microporous structures of the prepared PHA detected by Brunauer–Emmett–Teller. (B) Residual plasma protein concentration after PHA adsorption. (C) Plasma fibrinogen concentration after PHA adsorption. (D) The APTT, PT, TT, and INR tests using PHA-adsorbed plasma. (E) Plasma clotting factors concentration (in international units per deciliter) after PHA adsorbed. (F and G) MSB staining and SEM observation of manipulated fibrin films. (H) Semiquantitative analysis of fibrin film’s thickness. (I) Semiquantitative analysis of fibrin films porosity. (J) Residual plasma Ca^2+^ concentration. ns, not significant. *P < 0.05; **P < 0.01; ***P < 0.001; ****P < 0.0001.

We firstly proved that different groups of microporous structures were exposed differently through the Brunauer–Emmett–Teller analysis, especially at the range of 10 to 100 nm, whose incremental surface area and pore volume were larger in S group than those in M and L groups (Fig. 2A). Then, more adsorptions of blood proteins in S group were confirmed (Fig. 2B). There is no significant difference in fibrinogen among the groups (Fig. 2C), indicating that there was no difference in fibrinogen adsorption onto the materials. We then analyze several key proclotting factors using activated partial thromboplastin time (APTT), prothrombin time (PT), international normalized ratio (INR), and thrombin time (TT) tests to detect abnormal concentrations of blood clotting factors. PT tests the extrinsic coagulation pathway, and APTT tests the intrinsic pathway [43]. Prolonged APTT, PT, and INR of the surplus after adsorption in S group were observed, indicating that more intrinsic and extrinsic clotting factors XII, XI, IX, VIII, VII, and X were absorbed by S group (Fig. 2D), which were further confirmed (Fig. 2E). Thus, we successfully manipulated absorption of intrinsic and extrinsic clotting factors by tuning the PHA particle sizes. As fewer clotting factors were absorbed in L group, more clotting factors were in the remaining blood, which leads to increasing thickness and density of the surface fibrin films, whereas the S group did the opposite (Fig. 2F to I and Fig. S2). In addition, we also observed an interesting phenomenon that S group released more Ca^2+^ that may be due to its more microporous structure, while M and L groups adsorbed Ca^2+^ (Fig. 2J). There were many Ca^2+^ binding sites in the fibrin films, so there might be more Ca^2+^ binding in the fibrin films of S group.

Taken together, tuning the PHA particle sizes can manipulate the microporous structure, thus regulating the absorption of blood proteins and the subsequent formation of clot surface fibrin films. The fewer clotting factors adsorbed by microporous structure, the thicker and denser fibrin film formation on the surface. Our data show a remarkable aspect of blood–material interaction in which fibrin forms a manipulated film covering the external surface of biomaterials, suggesting that we should pay attention not only to the adsorption of blood protein but also to the fibrin films after implantation.

### The effects of generated fibrin film on FBR of macrophages and fibroblasts

Macrophages is the key driver and central orchestrator of fibrosis during FBR, attracting and inducing fibroblasts to the implant's surface and inducing their activation on arrival [9,44]. These activated fibroblasts adhere to the implant’s surrounding and begin depositing extracellular matrix proteins collagens, which cover the entire surface of the implants over time, leading to fibrous capsule [45–47]. Therefore, we focus on macrophage and fibroblast to investigate whether the generated fibrin films regulate them to mediate FBR.

**Fig. 3.**
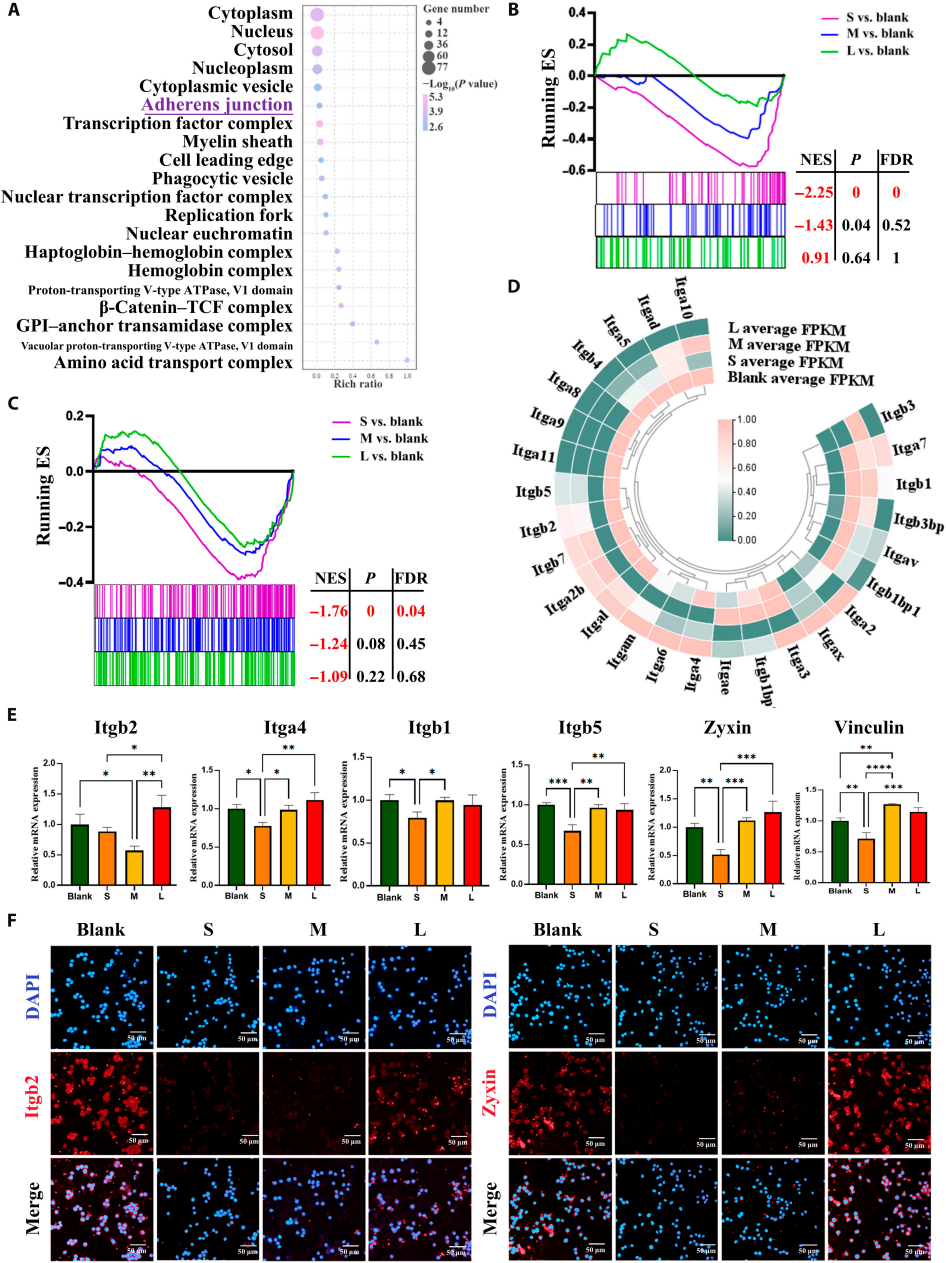
The adhesion behavior of macrophages is reduced on the thinner and sparser fibrin films. (A) Gene Ontology analysis shows the top 20 terms enriched in differentially genes for cellular component. (B and C) Gene Set Enrichment Analysis reveals the difference of adherens junction and focal adhesion. |Normalized enrichment score| (|NES|) > 1, nominal *P* < 0.05, and false discovery rate (FDR) *q* < 0.25 are considered significant. (D) Heatmap of cell adhesion related gene expressions. (E and F) RT-qPCR results of cell-adhesion-related gene expressions and representative immunofluorescence images of Itgb2 and Zyxin (classic adhesion marker) in macrophages cultured on fibrin films. *P < 0.05; **P < 0.01; ***P < 0.001; ****P < 0.0001.

Adhesion sequences and their interactions with cell adhesion receptors are crucial for macrophage recognition [48,49]. As previously mentioned, different fibrin films have different thicknesses and densities, which may regulate macrophages by cell adhesion behavior firstly. To test this hypothesis, we further used RNA sequencing (RNA-seq) to analyze the changes in gene expressions of macrophages. The analyses of the top 20 Gene Ontology terms showed that adherens junction was the most significant event from the 160 main differential genes (Fig. 3A and Fig. S3B). Adherens junction pathway significantly down-regulated in S group (Fig. 3B). All of cell adhesion related pathways were further screened, in which focal adhesion pathways were significantly enriched in all groups (Fig. S3C). Focal adhesion pathway significantly down-regulated in S group (Fig. 3C). These results suggest that cell adhesion (one of cell recognition) may be a major initial event when cells contact with fibrin films. Moreover, most of the detected adhesion genes and proteins were most significantly down-regulated in S groups, while L group was not obvious (Fig. 3D to F and Fig. S3D).

Interestingly, these results are not completely consistent with our previous studies on fibrin regulating macrophage adhesion behavior, which may be mainly due to biomaterial differences [50]. PHA contains Ca^2+^, which is a bivalent cation known to negatively regulate integrins [51–53]. PHA of S group released Ca^2+^, which increased Ca^2+^ binding on the fibrin films. Integrin of macrophages cultured on the fibrin films of S group was inhibited by high levels of Ca^2+^, so the cell adhesion level of S group decreased. While PHA of M and L groups adsorbed Ca^2+^, and Ca^2+^ binding on the fibrin films did not increase significantly. The integrin of macrophages cultured on the fibrin films of M and L groups was not inhibited by high level of Ca^2+^, so the cell adhesion level of M and L groups was not significantly decreased. At the same time, thinnest and sparsest fibrin films of S group reduce the number of cell adhesion sites, so the cell adhesion behavior of S group significantly inhibited.

**Fig. 4.**
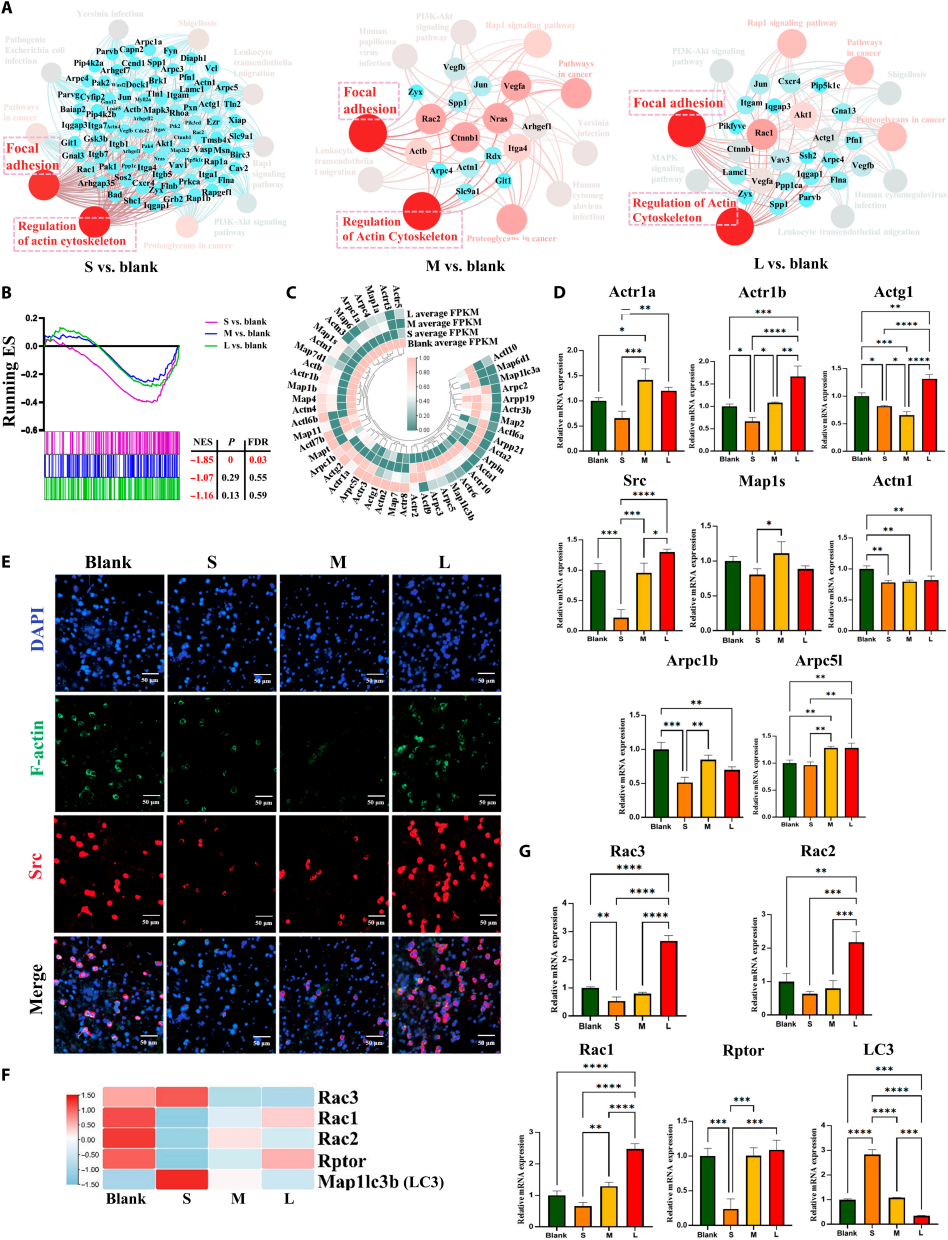
The adhesion-mediated cytoskeleton of macrophages is reduced on the thinner and sparser fibrin films. (A) Interaction network analysis showed close interaction between focal adhesion and regulation of actin cytoskeleton. (B) Gene Set Enrichment Analysis reveals the difference of regulation of actin cytoskeleton. |NES| > 1, nominal *P* < 0.05, and FDR *q* < 0.25 are considered significant. (C) Heatmap of cytoskeleton related gene expressions. (D and E) RT-qPCR results of cytoskeleton related gene expressions and representative immunofluorescence images of F-actin and Src (classic cytoskeleton marker) in macrophages cultured on fibrin films. (F and G) Heatmap and RT-qPCR results of cytoskeleton-accompanied autophagy-related gene expressions. *P < 0.05; **P < 0.01; ***P < 0.001; ****P < 0.0001.

The changes in intracellular functional events mediated by cell adhesion were further analyzed. We found that cell adhesion may mainly affect cytoskeleton (Fig. 4A and Fig. S4A). The regulation of actin cytoskeleton pathway was inhibited in 3 groups, and S group was the most significantly inhibited (Fig. 4B). Moreover, almost all cytoskeleton-related pathways were inhibited in the S group (Fig. S4B). Most of cytoskeleton-related genes and proteins were most significantly down-regulated in S groups (Fig. 4C to E and Fig. S4D). These results indicate that the change of cytoskeleton may be a major intracellular functional events after cells recognize fibrin films through cell adhesion. Poor cell-fibrin films adhesion leads to the inhibition of the cytoskeleton.

**Fig. 5.**
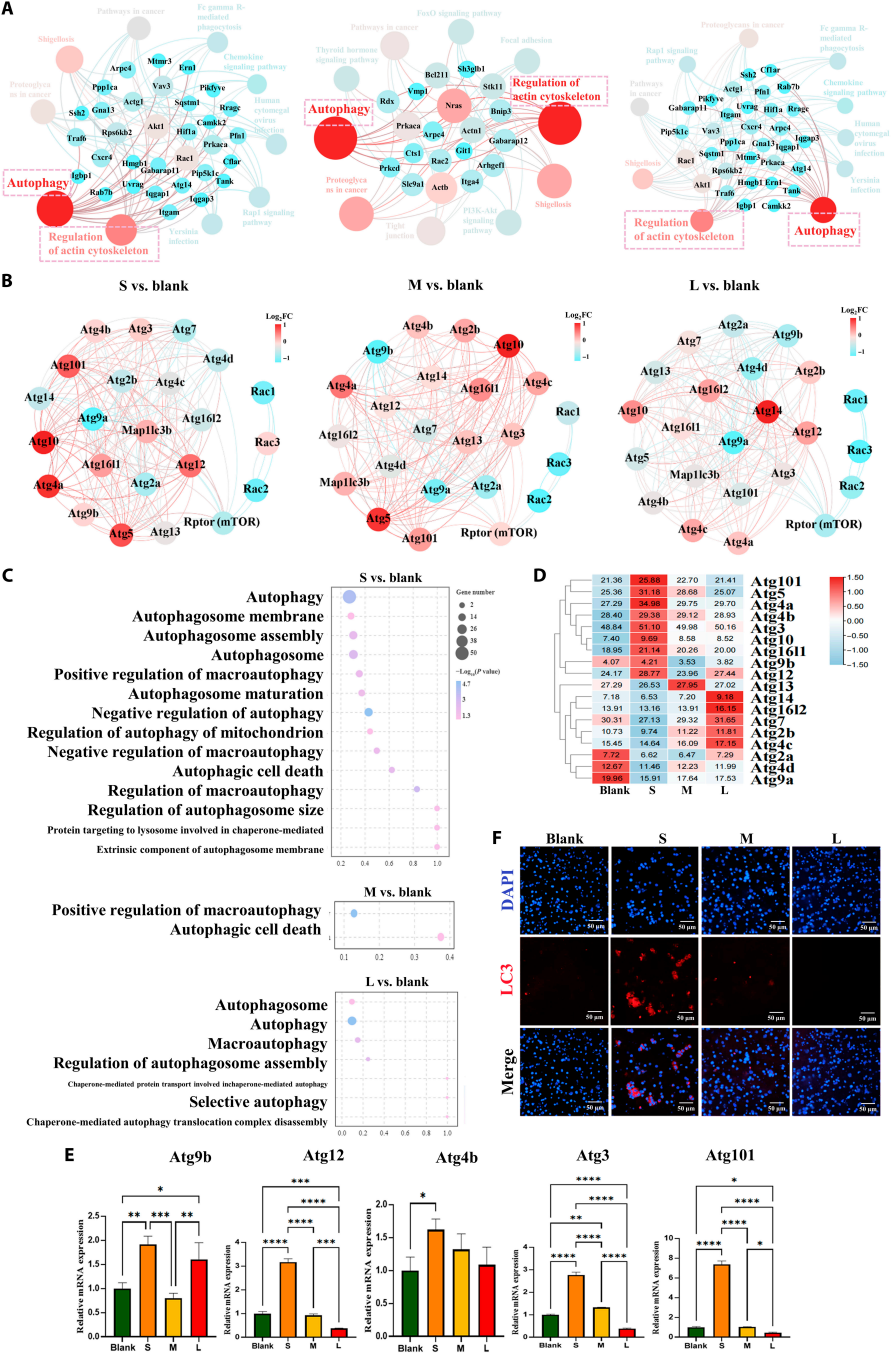
The cytoskeleton-accompanied autophagy of macrophages is increased on the thinner and sparser fibrin films. (A) Interaction network analysis showed close interaction between regulation of actin cytoskeleton and autophagy. (B) The Rac-mTOR pathway affected autophagy-related genes (Atgs) with negative regulation. (C) Autophagic-related pathways were enriched. (D) Heatmap of autophagy related gene expressions. (E and F) RT-qPCR results of autophagy-related gene expressions and representative immunofluorescence images of LC3 (classic autophagy marker) in macrophages cultured on fibrin films. FC, fold change. *P < 0.05; **P < 0.01; ***P < 0.001; ****P < 0.0001.

Autophagy is induced in a variety of cell types grown under low adhesion conditions [54]. The reduction of adhesion-related protein integrin induces autophagy in cells [55]. Detachment-induced autophagy directly results from the reduction of adhesion-related protein integrin. Fibrin films serves as the adhesion matrix of macrophages, the reduction of fibrin film adhesion site, and adhesion-related protein and genes in S group. These lead to poor cell adhesion, and the low adhesion integrin level may induce autophagy. Almost all cytoskeleton-related pathways, genes, and proteins were down-regulated by the poor cell adhesion, but we observed that microtubule-associated protein 1A/1B light chain 3B (LC3) (a central protein in autophagy) was unexpectedly up-regulated in S group (Fig. 4F and G) [56,57]. These results indicated that the poor cell adhesion in S group induces increased autophagy. Besides, Racs and Rptor [regulatory-associated protein of mammalian target of rapamycin (mTOR), negative regulator of autophagy] expressions were down-regulated in S group (Fig. 4F and G and Fig. S4C) [58–60]. Network interaction analysis further proved that cytoskeleton and autophagy have an interrelationship and the Racs-mTOR pathway affected autophagy-related genes with negative regulation (Fig. 5A and B). These results may imply that inhibition of cytoskeleton is accompanied by the activation of autophagy. Moreover, almost all autophagy-related pathways were screened, and autophagy-related pathways were significantly enriched in the S group (Fig. 5C). Most of autophagy-related genes and proteins were the most significantly up-regulation (Fig. 5D to F and Fig. S3D) contrary to the down-regulation of cytoskeleton in S group. These results confirmed that the poor cell adhesion in S group induces increased autophagy.

**Fig. 6.**
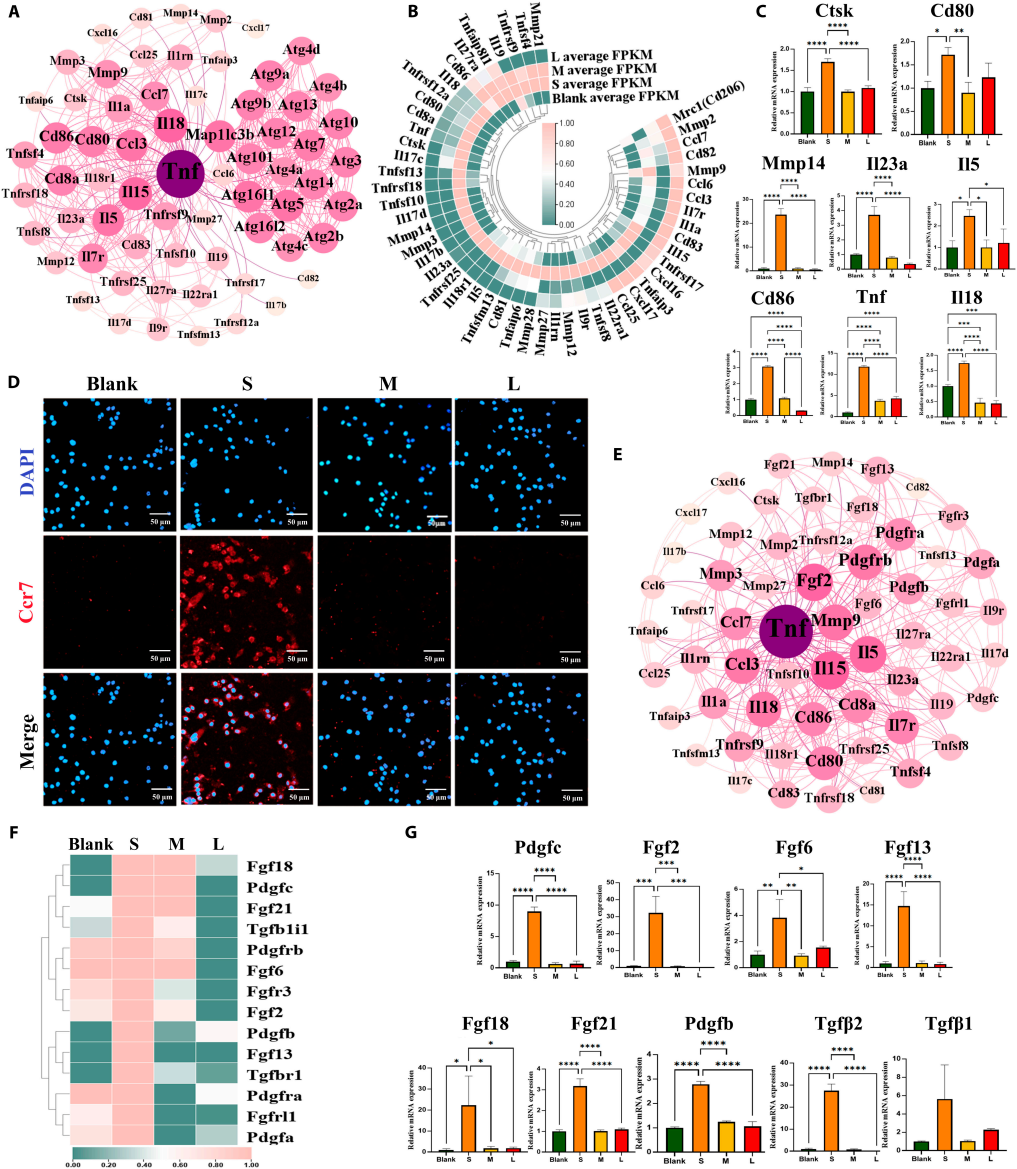
The autophagy-mediated inflammation and profibrosis of macrophages are increased on the thinner and sparser fibrin films. (A) Interaction network analysis showed close interaction between autophagy-related genes and inflammatory-related genes. (B and C) Heatmap and RT-qPCR results of inflammatory related gene expressions. (D) Representative immunofluorescence images of CCR7 (classic inflammation marker) in macrophages cultured on fibrin films. (E) Interaction network analysis showed close interaction between inflammatory-related genes and profibrosis-related genes. (F and G) Heatmap and RT-qPCR results of profibrosis-related gene expressions. *P < 0.05; **P < 0.01; ***P < 0.001; ****P < 0.0001.

Mild autophagy ensures a well-balanced inflammatory response, but excessive autophagy may lead to excessive inflammation [61–63]. The results of network interaction showed that there was an interaction between inflammatory-related genes and autophagic genes (Fig. 6A). Most inflammatory genes and proteins in S group were significantly up-regulated (Fig. 6B to D and Fig. S3D), which implies that excessive autophagy may enhance the inflammatory response. Fibrosis is the end-products of inflammatory response in FBR processes [64]. The results of network interaction indicated that there was an interaction between inflammatory-related genes and profibrosis genes (Fig. 6E). Most of the profibrosis genes were significantly up-regulated in the S group (Fig. 6F and G), which implies that excessive inflammation may enhance fibrosis. These results indicated that excessive autophagy leads to enhanced inflammatory response and secretion of profibrosis factors.

**Fig. 7.**
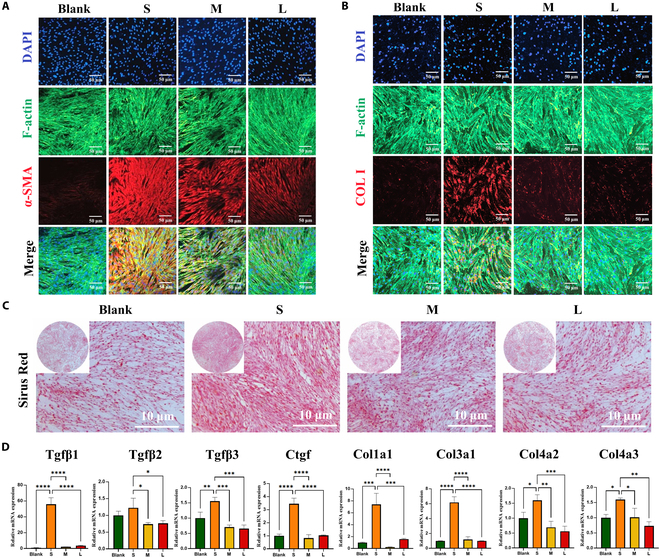
Activated macrophage regulates the collagen secretion and collagen matrix formation of fibroblasts. (A and B) Representative immunofluorescence images of α-SMA (activated fibroblast marker) and COL-I (collagen I marker) in fibroblasts cultured in the conditional medium from activated macrophages on fibrin films. (C) Representative images of collagen matrix formation by fibroblasts. (D) RT-qPCR results of collagen secretion and collagen-matrix-formation-related gene expressions. *P < 0.05; **P < 0.01; ***P < 0.001; ****P < 0.0001.

To further evaluate the effect of these inflammatory and profibrosis cytokines secreted by macrophages on FBR, we collected and analyzed fibroblasts cultured in the conditioned medium of macrophages on fibrin films. The results showed that the fibroblasts were activated significantly (Fig. 7A and Fig. S3D) and secreted more collagen and collagen matrix (Fig. 7B and C and Fig. S3D), and the expression of fibrosis-related gene was significantly up-regulated in S group (Fig. 7D). These results indicated that fibrin films indirectly regulate collagen secretion and collagen matrix formation of fibroblasts by regulating macrophages.

Taken together, S group formed thinner and sparser fibrin films with a poor cell adhesion, inhibited cytoskeleton, and enhanced autophagy, leading to stronger inflammation, collagen secretion, and collagen matrix formation. However, L group formed thicker and denser fibrin films with a better cell adhesion, uninhibited cytoskeleton, and unincreased autophagy, leading to more moderate inflammation, collagen secretion, and collagen matrix formation. These results prove that fibrin films affect inflammation and profibrosis of macrophages through a potential pathway of cell adhesion–cytoskeleton–autophagy and ultimately affecting fibroblast-mediated collagen secretion and collagen matrix formation.

### The functions of generated fibrin film on the FBR in vivo

**Fig. 8.**
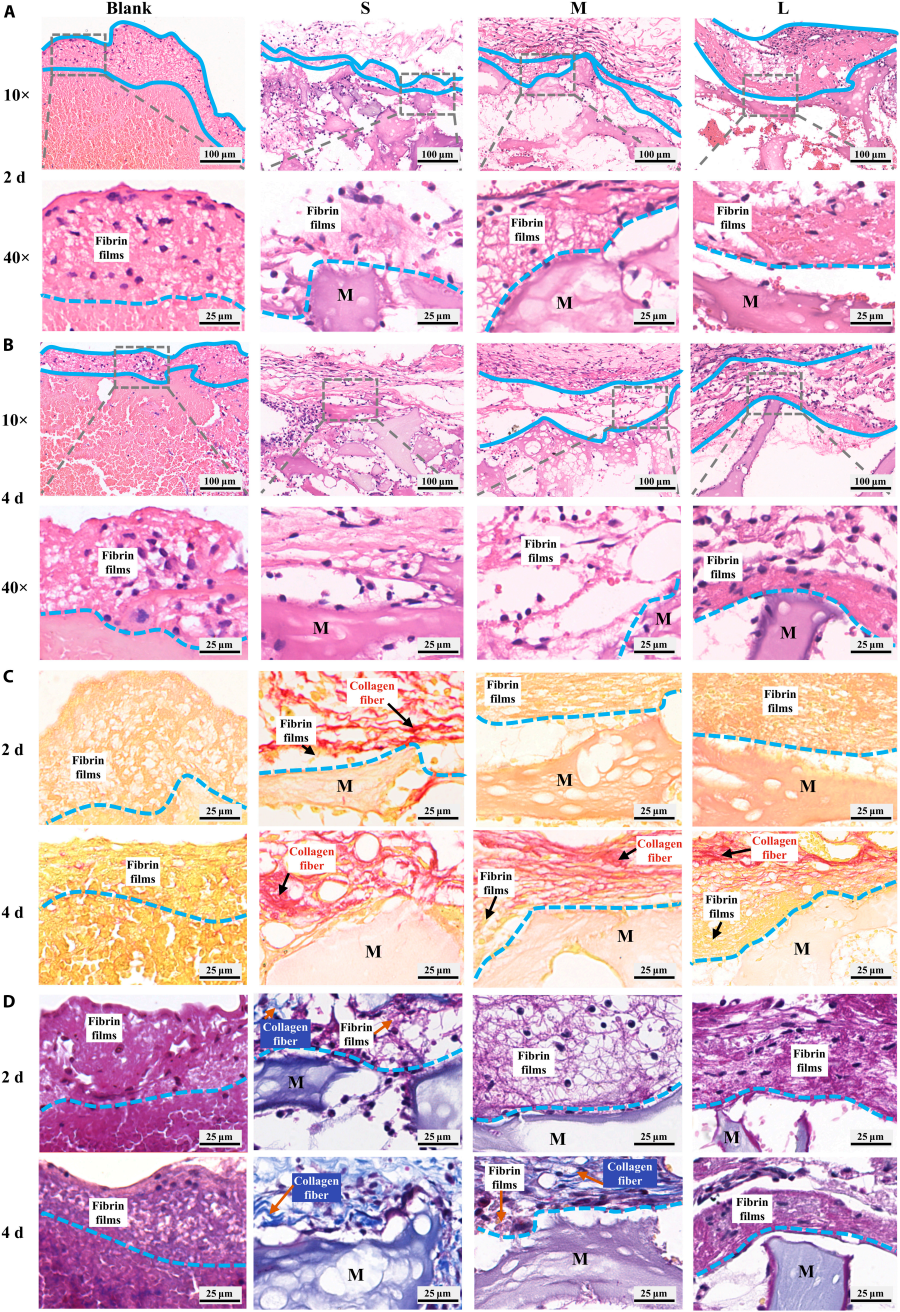
Fibrin films observed in vivo. (A and B) Hematoxylin and eosin staining showed a films (blue dashed line) covering the surface of PHA. (C) Sirius red staining showed this films (golden yellow) was not collagen fiber (red). (D) MSB staining showed this films was fibrin films (purple red), not collagen fiber (dark blue). M, materials.

After verifying that fibrin films can regulate the FBR mediated by macrophages and fibroblasts in vitro, we further confirmed fibrin films mediating FBR in vivo. We observed a “films” with different thicknesses covering the surface of PHA particles, and they started to degrade on 4 d after implantation. The fibrin film’s thickness of S group was the thinnest on 2 d and completely degraded on 4 d (Fig. 8A and B and Fig. S5C). Sirius Red and Martius Scarlet Blue (MSB) staining results showed that this films were not collagenous fiber, but fibrin films (Fig. 8C and D). These results indicate that the thickness and degradation time of surface fibrin film were different, which were affected by sizes of PHA particles.

**Fig. 9.**
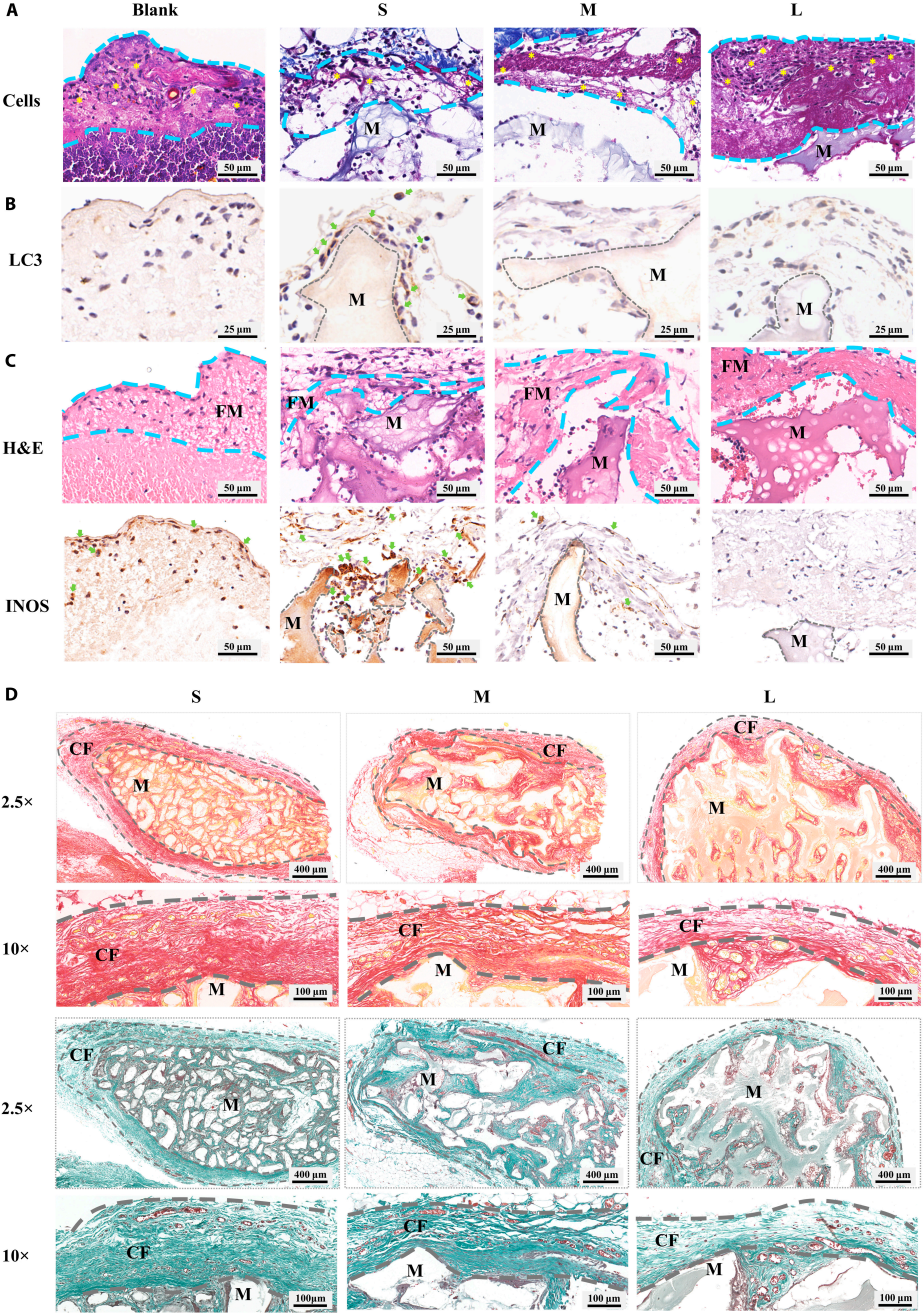
The functions of generated fibrin film on the FBR in vivo. (A) After 2 d, MSB staining showed cells (yellow asterisk) adhered to the fibrin films (blue dashed line). (B) After 2 d, LC3 (classic autophagy marker, green arrow) staining showed increased autophagy on the thinner and sparser fibrin films. (C) After 2 d, hematoxylin and eosin (H&E) and INOS (inflammatory macrophage marker, green arrow) staining showed increased inflammation on the thinner and sparser fibrin films. (D) After 4 weeks, Sirius Red and Goldner staining showed the increased fibrous capsule (gray dashed line) and collagen deposition on the thinner and sparser fibrin films. FM, fibrin films; CF, collagen fiber.

Then, we observed many cells in the fibrin films (Fig. 9A and Fig. S5A), suggesting that the first step for cells to recognize biomaterials may be contact fibrin films, which were consistent with previously described in vitro results. CD68 (macrophage marker) and α-smooth muscle actin (α-SMA) (fibroblast marker) staining results further showed macrophages and fibroblasts on the fibrin films (Fig. S5B), suggesting that there is cross-talk between macrophages and fibroblasts on fibrin films.

The underlying mechanism of fibrin films affecting FBR was further confirmed. The cell adhesion was the least, but autophagy, inflammation, and fibrosis were the strongest in S group; while the cell adhesion was the most, but autophagy, inflammation, and fibrosis were more moderate in L group (Fig. 9 and Fig. S5C and D). These results suggested that fibrin films regulated FBR through potential signaling pathways of cell adhesion, autophagy, inflammation, and fibrosis, which were consistent with previously described in vitro results.

Taken together, PHA particle size affects the thickness and degradation of fibrin films. The thinner and sparser fibrin films in S group performed poor cell adhesion and enhanced autophagy, leading to stronger inflammation and fibrosis. However, the thicker and denser fibrin films in L group performed better cell adhesion and unincreased autophagy, leading to more moderate inflammation and fibrosis. These results prove that fibrin films can separate the biomaterials from surrounding tissue and keep the implanted biomaterial–blood clot mixture into a relatively isolated lesion environment. This prevents the surrounding tissue and cells from directly interacting with foreign biomaterials, regulating macrophages, and fibroblasts, leading to more moderate FBR, and demonstrating a bioprotective effect.

### Implications for the tuning of FBR

As a vital initial event in the entire cell recognition process and cell responses to implantable biomaterials, fibrin film elicits vital effects on determining the eventual outcomes of FBR. However, most of the current knowledge for implantable biomaterials directly regulates the surface protein adsorption-mediated FBR, while ignoring the effect of fibrin films formed on the biomaterial surface after protein adsorption. This goes against the natural series of events of FBR rule that starting events skip the intermediate stage and directly determine the subsequent consecutive events. These could cause some unexpected in vitro and in vivo results and a lower clinical success rate. Therefore, it is still important to deepen the understanding of cells recognition and cell responses to biomaterials in FBR for mitigating fibrosis outcomes.

It should be noted that fibrin film has a number of parameters that may be involved in cells recognition and cell responses in FBR, including density, thickness, pore size, porosity, mechanical strength, and degradation pattern. The change of fibrin film’s thickness and density is usually accompanied by a number of changes such as porosity, pore size, degradation, mechanical strength, etc. This thickness–density coupling regulation of fibrin films can become an effective strategy to control cell recognition and cell responses to reduce FBR strength. Future studies need to further study the effects of changes in porosity, pore size, degradation, mechanical strength, and other changes accompanied by changes in fibrin film’s thickness and density on cell behavior. Only in this way can we better understand the role of fibrin films in FBR, so as to provide an optimal solution to the problem of FBR in biological materials.

In addition, it can also be observed that the thickness of fibrin films on the surface of PHA particles was not uniform. Even in the blank group (the simple blood clot group without adding any material), the thickness of the fibrin films formed on the surface is not uniform. Although the thickness of fibrin films and the inflammation level by all sites were comprehensively evaluated, the uneven thickness of fibrin films on the surface of PHA particles at different sites may lead to different localized inflammatory responses. This leads to the speculation that whether the fibrin film can be uniformly tuned. To achieve this aim, the formation of blood clot should be thoroughly understood. During the formation of a blood clot, contraction occurs to form fibrin films. Shrinkage of blood clots occurs because of blood intracellular traction forces adhering to fibrin fibers, which form the viscoelastic 3-dimensional framework of clots and distribute traction forces throughout them [65]. The contraction of blood clots is a spatially heterogeneous process, with asymmetry contractile force because the contractile forces acting on the periphery are not compensated and drastic differences in the speed at the edge of the contracting clot and inside the clot such that the peripheral part of the macroscopic clot contracts faster and the shrinkage propagates toward the center [66]. Because of the uneven contraction speed of the blood clot, the thickness of the fibrin films formed by coagulation contraction will also be anisotropy.

**Fig. 10.**
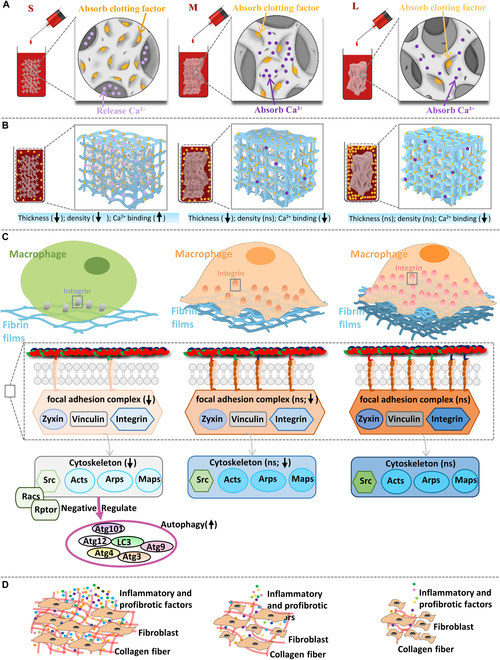
Schematic figure of the underlying mechanisms of PHA microporous structures regulating blood clot fibrin films to manipulate biomaterial-mediated FBR. (A) PHA with different numbers of micropore structures, resulting in different clotting factor absorptions and Ca^2+^ release. (B) The residual clotting factor and Ca^2+^ in the blood cause different thicknesses, densities, and Ca^2+^ binding of fibrin films. (C) Fibrin films of different thicknesses, densities, and Ca^2+^ binding affect adhesion-related protein quantity and conformation to further regulate intracellular signaling. (D) Macrophages regulated by fibrin films produce different levels of inflammatory and profibrotic factors to regulate the collagen secretion and collagen matrix of fibroblasts, leading to FBR.

Therefore, to control the consistency of the fibrin film’s thickness, the following factors need to be at least controlled. First of all, it is necessary to control the uniformity and balance of clot contractile force in all directions to reduce the anisotropic heterogeneity of coagulation contraction, which is crucial to the consistency of the fibrin film’s thickness. Second, it is necessary to control the consistency of clot contraction speed in all directions to make the consistency of the speed of inward contraction in all directions, which is crucial for the consistency of fibrin film’s thickness. In addition, it is also necessary to control the uniformity of the material, including the physical properties (shape, size, pore size, porosity, etc.) and chemical properties (surface charge, pH, ion release, etc.), so that the factors of the material in the formation of the fibrin film are uniform, so as to obtain the thickness uniformity of the fibrin film controlled by the material. All these require more efforts to deeply reveal the specific mechanisms that affect clot contractile force and speed and reach a consensus to better apply to the research field of the interaction between biological materials and blood and achieve precise regulation of surface fibrin film’s thickness. Although fibrin film formation was a complicated and unrevealed process, it is believed that it is related to the amounts of clotting factors and fibrinogen. We regulated the amounts of clotting factors and then succeeded in regulating this fibrin films, which indicates the feasibility of tuning fibrin films for controlling FBR (Fig. 10).

How to adjust the physicochemical property of biomaterials to regulate fibrin films is an interesting subject, which may provide another improved aspect for the controlling of FBR strength via the coupling regulation of protein absorption and fibrin films. Fibrin films play a key role in cell recognition and cell responses during FBR processes, which coincides with the growing importance of fibrin films. However, our understanding of fibrin film formation and its regulatory FBR remains superficial. Although we explored that the regulatory role of clotting factors amounts on fibrin film formation and fibrin films on macrophages and fibroblasts in this study, other clotting components and immune cells are also involved in the fibrin film formation and FBR process, respectively. The key to solving this problem is a comprehensive and in-depth understanding of the fibrin film formation and its role in initiating the cellular response in FBR, which is still poorly studied. We can only comprehend it better if we deepen our understanding.

## Materials and Methods

### Preparation and characterization of PHA with different sizes

PHA were prepared according to our early study [34]. The cancellous bone derived from the porcine femoral was immersed in 30% hydrogen peroxide and anhydrous ethanol, then divided into blocks, calcinated in air in a muffle furnace, and pulverized into powders with 3 different particle sizes: <0.2,1 to 2, and 3 to 4 mm. The surface and dimensions of the PHA particles were observed and verified by stereomicroscopy (Leica, Germany). The surface morphological characteristics of PHA were observed by SEM (JEOL, Japan). The samples were tested by an x-ray powder diffractometer. The infrared spectra were collected by Fourier transform infrared (Ettlingen, Germany).

### Clotting factor adsorption

The pore sizes were detected using the nitrogen adsorption and desorption instrument (Mike ASAP2460, USA). PHA was mixed with plasma diluted twice with ultrapure water and centrifuged at 1,000 rpm for 1 min. Protein concentration in the supernatant was detected by bicinchoninic acid assay protein assay (Cwbiotech, China). APTT, PT, TT, INR, fibrinogen, and clotting factors in supernatant were measured (KingMed Diagnostics, China). Calcium ion concentration determination with supernatant (Agilent 7700,USA).

### Fibrin films characterization

The blood clots fibrin films were prepared by mixing PHA and rat blood in 2 h. The blank group had no PHA. The single fibrin films were prepared by mixing PHA, blood plasma, and thrombin reagent (Haifei, China) in 2 h. Fibrin films displayed by MSB staining. For the morphological observation in SEM, the fibrin films were soaked in a 2.5% glutaraldehyde solution to fixation, followed by graded ethanol, solvent replacement, and lyophilization. The porosity of the fibrin films was calculated using ImageJ software, and the thickness of the fibrin films was calculated using ImageScope software.

### Macrophages cultured on fibrin films

Dulbecco’s modified Eagle medium (Gibco, USA) with 10% fetal bovine serum (Gibco) and 1% penicillin/streptomycin (Gibco) was used as the medium to culture RAW 264.7 cell line. Place culture flasks in a cell incubator (37 °C at 5% CO_2_) and replaced the medium every 2 d. The cells passaged after 80% convergence. Fibrin films were extracted, and macrophages were seeded and cultured for 48 h. The conditioned media were collected for further fibroblast stimulation. The cultured macrophages were used for RNA-seq, reverse transcription quantitative polymerase chain reaction (RT-qPCR) and immunofluorescence staining. RNA-seq was performed using the BGISEQ-500 platform at BGI (Wuhan, China). Raw data of FASTQ format were calculated into read counts and FPKM (fragments per kilobase of exon model per million mapped fragments) data for further analysis. Principal components analysis of transcriptome data showed good intragroup repeatability and between-group variability (Fig. S3A). All data visualization was performed by GraphPad Prism (v8.0.1), Gephi (v0.9.2), TB tools (v1.095), and Omic Share tools (https://www.omicshare.com/tools).

### Isolation of primary human gingival fibroblasts and stimulation

Human gingival fibroblasts were extracted with the patient’s written informed consent and approved by the Ethics Review Committee of the Guanghua School of Stomatology, Sun Yat-sen University, China (no. KQEC-2019-06-02). Healthy gum samples from patients with tooth extraction. The tissue was digested with Protease disease II (Sigma-Aldrich, USA) and cut into small pieces. Gingival connective tissue tablets were planted on a Dulbecco’s modified Eagle medium culture vial containing 15% fetal bovine serum. The fibroblasts were extracted from the gingival tissue and cultured until the fusion reached 80% and then replaced with macrophage-conditioned medium for further stimulation.

### RT-qPCR and immunofluorescence staining

The extracted total RNA was detected by Nanodrop (Thermo Fisher Scientific, USA) and then converted to complementary DNA by PrimeScript RT Master Mix (Takara, Japan). Equivalent cDNA samples were analyzed by RT-qPCR analysis using Hieff qPCR SYBR Green Master Mix (Yeasen, China) on an ABI 2-step system (Applied Biosystems, USA). Primer sequences were shown in the Supplementary Materials (Tables S1 and S2). The obtained samples were calculated based on the 2^−ΔΔCt^ method referring to glyceraldehyde-3-phosphate dehydrogenase. Macrophages were stained by 4′,6-diamidino-2-phenylindole simultaneously with integrin β_2_ (1:100; Proteintech), Zyxin (1:500; Abcam), Src (1:100; Proteintech), LC3 (1:100; Cell Signaling Technology), and CCR7 (1:100; Abcam). Fibroblasts were stained by actin, 4′,6-diamidino-2-phenylindole, α-SMA (1:100; Affbiotech), and collagen I (1:100; Abcam) antibody. Alexa Fluor 594 goat anti-mouse secondary antibody (Emarbio, China) and Alexa Fluor 647 goat anti-rabbit secondary antibody (Beyotime, China) were used for different purposes.

### Animal surgery and sample preparation

Male Sprague–Dawley rats from the Experimental Animal Center of Sun Yat-sen University [SYXK (Guangdong) 2017-0081] were used as the experimental animals. All experiments involving animal use were approved by the Institutional Animal Care and Use Committee of Sun Yat-sen University (approval no: SYSU-IACUC-2019-000058). Animal surgery and sample preparation were performed under general anesthesia (Zoletil 50, 0.2 ml/100 g). Four vertical subcutaneous incisions (about 1cm) were performed on the back of rats. After hemostasis was stopped by applying cotton ball pressure, the subcutaneous tissues on both sides were bluntly separated (blunt separation could avoid blood vessel injury and bleeding), and blood prefabricated PHA or blood clot (blank group)was implanted subcutaneously on both sides of the back about 2 cm away from the incision. The animals were sacrificed after 2 d, 4 d, and 4 weeks, and subcutaneous samples were harvested.

### Histological analysis

Tissue samples fixed in 4% paraformaldehyde solution that buffered by 0.1 M phosphate solution for 24 h and then decalcified in 4% EDTA for 4 weeks. Tissue samples were stained by Mayer’s hematoxylin (Servicebio, China) and eosin (Servicebio, China), Goldner, Sirius Red, MSB (Pythonbio, China), CD68 antibody (1:100; Abcam), inducible nitric oxide synthase (iNOS) antibody (1:50; Abcam), α-SMA antibody (1:100; Affbiotech), LC3 antibody (1:100; Cell Signaling Technology), and goat anti-mouse/rabbit secondary antibody (GeneTech, China) for different purposes. Images were captured using Aperio AT2 system (Leica Aperio AT2, Germany) and semiquantification was conducted using ImageJ software (1.46) and ImageScope software.

### Statistical analysis

All experiment was repeated at least 3 times independently. The representative SEM, immunofluorescence, histological section staining image were presented. All the experimental results were expressed as means ± SD. One-way (for one independent variable) analysis of variance (ANOVA) followed by Tukey’s multiple comparison post hoc test was conducted unless otherwise stated. Statistical analysis of data performed by GraphPad Prism (v 8.0.1). In all cases, *P* value below 0.05 considered statistically significant (*P < 0.05; **P < 0.01; ***P < 0.001; ****P < 0.0001).

## Data Availability

The raw/processed data required to reproduce these results cannot be shared at this time as the data also form the part of an ongoing study.
