# Supplementary material for: Regulating Blood Clot Fibrin Films to Manipulate Biomaterial-Mediated Foreign Body Responses
Source: Research (Wash D C). 2023 Sep 15;6:0225. doi: 10.34133/research.0225 (PMC10503960; doi:10.34133/research.0225)
Supplement: Supplementary 1 — Figs. S1 to S5 Tables S1 and S2 [file research.0225.f1.zip › Supplementary Materials(Figure Legend).docx]

**Supplementary Materials (Figure Legend)**

**Fig. S1.** Physiochemical characterization of PHA particles. (A) The stereomicroscopic images showed PHA particles with three different sizes were well-controlled. (B) XRD patterns of PHA particles and FTIR spectra of chemical groups were matched with the hydroxyapatite. (C) SEM images of different size PHA particles show that their biomimic microporous structure.

**Fig. S2.** SEM observation of manipulated fibrin films.

**Fig. S3.** (A) Principal Component Analysis (PCA) of transcriptome data of macrophages on different fibrin films. (B) Venn diagram shows differential gene expression of macrophages on different fibrin films. (C) Focal adhesion pathways were significantly enriched in all groups. (D) Semi-quantitative analysis.

**Fig. S4.** (A) Interaction network analysis showed close interaction between Focal adhesion and Regulation of actin cytoskeleton. (B) almost all cytoskeleton-related pathways were inhibited on the thinner and sparser fibrin films. (C) Inhibition of Rac-mTOR pathway leads to enhanced autophagy.

**Fig. S5.** (A) HE staining showed many cells on the fibrin films (blue dotted line). (B) CD68 (macrophage marker, green arrow) and α-SMA (fibroblast marker, green arrow) staining showed macrophages and fibroblasts on the fibrin films. (C) Semi-quantitative analysis. (D) HE and INOS (inflammatory macrophage marker, green arrow) staining showed increased inflammation on the thinner and sparser fibrin films on 4 days. M: materials; FM: fibrin films.
