# Supplementary material for: Regulating Blood Clot Fibrin Films to Manipulate Biomaterial-Mediated Foreign Body Responses
Source: Research (Wash D C). 2023 Sep 15;6:0225. doi: 10.34133/research.0225 (PMC10503960; doi:10.34133/research.0225)
Supplement: Supplementary 1 — Figs. S1 to S5 Tables S1 and S2 [file research.0225.f1.zip › Table S1.pdf]

Table S1. Primer sequences for macrophages

Gene	Primer sequences
Itgb2	Forward: 5’-GGAGTCACCTGCTCCTTCTCTC-3’
	Reverse: 5’-TTGGTGCATTCCTGGGACAC-3’
Itga4	Forward: 5’-AGACGTGCGAGACATCCTTAC-3’
	Reverse: 5’-CCAGCGTTGAACAAGGACAC-3’
Itgb5	Forward: 5’-CAGCTACACAGAACTGCCCA-3’
	Reverse: 5’-CTGGAGCGCTCACTTTGGAA-3’
Vinculin	Forward: 5’-TGGTCTAGCAAGGGCAATGA-3’
	Reverse: 5’-TGATGCACAGCCCAGAGAAG-3’
Zyxin	Forward: 5’-TCTGTGCTGCTAGGTTGTGG-3’
	Reverse: 5’-CCCTTGATGTTTTTCCCCGT-3’
Atg9b	Forward: 5’-AGTGCTGTTGTTGTCCTGCTC-3’
	Reverse: 5’-CCGTTCGTCTCACCATCAGG-3’
Atg101	Forward: 5’-GACTCGGTCCTCGTCGTCT-3’
	Reverse: 5’-CCGAGAATTCTGACCGGACC-3’
Atg12	Forward: 5’-CACACATGGCAGCACTCCTA-3’
	Reverse: 5’-TTCCCCCAGAGGTGAGACAA-3’
Atg4b	Forward: 5’-TCCAGCTATTGATTGGAGGTGG-3’
	Reverse: 5’-GGGACTGGGGCATCATGAAA-3’
Atg3	Forward: 5’-GTGAAGGGAAAGGCTCTGGAA-3’
	Reverse: 5’-TAAGTGATCTCCAGCTGCCAC-3’
Actn1	Forward: 5’-TGATATTGGCAACGACCCCC-3’
	Reverse: 5’-AGCGGTTGGGGTCTACAATG-3’
Actr1a	Forward: 5’-TGTTCAAAGATATCTGCACCCCA-3’
	Reverse: 5’-CGTCACCATCGTGGGTAGTT -3’
Actr1b	Forward: 5’-TCGACAATGGTTCAGGGGTG-3’
	Reverse: 5’-CAATGAACAGGTCCCCCTCC-3’
Actg1	Forward: 5’-TCGAACACGGCATTGTCACT-3’
	Reverse: 5’-ACATTATCTGCGTCATCTTCTCT-3’
Arpc5l	Forward: 5’-AACACACTGTCCTCACGCTT-3’
	Reverse: 5’-AGGAGCCCGTCTACCTCG-3’
Arpc1b	Forward: 5’-GCACAAGAACAGTGTCAGCC-3’
	Reverse: 5’-TTAGCAAAGCAGCCCTTAGCC-3’
Map1s	Forward: 5’-GTCATGGAGGAGTTGGAACGA-3’
	Reverse:5’-CTCCGCTGCCCTTTCACAA-3’
Map1lc3b	Forward: 5’-AGATCCCAGTGATTATAGAGCGA-3’
	Reverse: 5’-ACTTCGGAGATGGGAGTGGA-3’
Rac3	Forward: 5’-TTCGGCCACTCTCCTATCCT-3’
	Reverse: 5’-GACGGAACCAATCTCTCGGG-3’

Rac2	Forward: 5’-GATGCAGCCATCCATCCTCA-3’
	Reverse: 5’-AGAGACAGTAGGGGTTGGCT-3’
Rac1	Forward: 5’-GAGAGTACATCCCCACCGTC-3’
	Reverse: 5’-CGGCAATCGGCTTGTCTTT-3’
Rptor	Forward: 5’-CTGCCCTTGCCAGATGAGAA-3’
	Reverse: 5’-TGGCAACCAGCATGTCTTCA-3’
TNF	Forward: 5’-GATCGGTCCCCAAAGGGATG-3’
	Reverse: 5’-GGTTTGCTACGACGTGGGC-3’
IL18	Forward: 5’-TACAAGCATCCAGGCACAGC-3’
	Reverse: 5’-CTGATGCTGGAGGTTGCAGA-3’
IL5	Forward: 5’-AGCAATGAGACGATGAGGCT-3’
	Reverse: 5’-AGCATTTCCACAGTACCCCC-3’
Mmp14	Forward: 5’-AGGCCAATGTTCGGAGGAAG-3’
	Reverse: 5’-TCAAAGGGTGTACTGTCGCC -3’
Cd80	Forward: 5’-AGTTTCTCTTTTTCAGGTTGTGAA-3’
	Reverse: 5’-ACATGATGGGGAAAGCCAGG-3’
Cd86	Forward: 5’-CTTACGGAAGCACCCACGAT-3’
	Reverse: 5’-TGTAAATGGGCACGGCAGAT-3’
Il23a	Forward: 5’-TGGAGCAACTTCACACCTCC-3’
	Reverse: 5’-GGCAGCTATGGCCAAAAAGG-3’
Ctsk	Forward: 5’-CCTGTTGGGCTTTCAGCTCT-3’
	Reverse: 5’-CCGTTCTGCTGCACGTATTG-3’
Fgf2	Forward: 5’-GGCTGCTGGCTTCTAAGTGT-3’
	Reverse: 5’-TTCTGTCCAGGTCCCGTTTT-3’
Fgf6	Forward: 5’-GGTGTGAATTGGGAAAGCGG-3’
	Reverse: 5’-TAGGGGTTCTCCTCGTGTGT-3’
Fgf13	Forward: 5’-ATGCTTCTAAGGAGCCTCAGCTT-3’
	Reverse: 5’-TTGCTGCTGACGGTAGATCA-3’
Fgf18	Forward: 5’-GGACCAGTGGGAAGCACATT-3’
	Reverse: 5’-CGAGCTTGCCTTTTCGGTTC-3’
Fgf21	Forward: 5’-ACGACCAAGACACTGAAGCC-3’
	Reverse: 5’-GGAGACTTTCTGGACTGCGG-3’
Pdgfb	Forward: 5’-CTTTCTCCAGAGAGATATTTTGCG-3’
	Reverse: 5’-TTGCAACGAAGCTAGAGGGTG-3’
Pdgfc	Forward: 5’-AAGGAACAGAACGGAGTGCAA-3’
	Reverse: 5’-TGAGGAAACTTCGGGCTGTG-3’
Tgfβ1	Forward: 5’-CATCCATGACATGAACCGGC-3’
	Reverse: 5’-GAAGTTGGCATGGTAGCCCT-3’
Tgfβ2	Forward: 5’-TTACAACACCCTCTGGCTCATTG-3’
	Reverse: 5’-TTAGCAGGAGATGTGGGGTCT-3’

Itgb1	Forward: 5'-ATGCCAAATCTTGCGGAGAAT-3'
	Reverse: 5'-TTTGCTGCGATTGGTGACATT-3'
Src	Forward: 5'-GCTCTTCGGAGGCTTCAACT-3'
	Reverse: 5'-CTGACATCCACCTTCCTCGT-3'
GAPDH	Forward: 5'-AGGTCGGTGTGAACGGATTTG-3'
	Reverse: 5'-TGTAGACCATGTAGTTGAGGTCA-3'
