# Supplementary material for: Regulating Blood Clot Fibrin Films to Manipulate Biomaterial-Mediated Foreign Body Responses
Source: Research (Wash D C). 2023 Sep 15;6:0225. doi: 10.34133/research.0225 (PMC10503960; doi:10.34133/research.0225)
Supplement: Supplementary 1 — Figs. S1 to S5 Tables S1 and S2 [file research.0225.f1.zip › Table S2.pdf]

Table S2. Primer sequences for fibroblasts

Gene	Primer sequences
Col1a2	Forward: 5’-GGATGAGGAGACTGGCAACC-3’
	Reverse: 5’-TGCCCTCAGCAACAAGTTCA-3’
Col3a1	Forward: 5’-CGCCCTCCTAATGGTCAAGG-3’
	Reverse: 5’-TTCTGAGGACCAGTAGGGCA-3’
Col4a2	Forward: 5’-GGACAGACGAGACAACAGCA-3’
	Reverse: 5’-GAGCTGGCATAACATTGGCG-3’
Col4a3	Forward: 5’-CCTAGGGGAGAGCTCAGTGT-3’
	Reverse: 5’-TTCCTGGTTCACCATCAGGC-3’
Ctgf	Forward: 5’-GTGGAGTATGTACCGACGGC-3’
	Reverse: 5’-GCAGGCACAGGTCTTGATGA-3’
Tgfβ1	Forward: 5’-CAAGACCACCCACCTTCTGG-3’
	Reverse: 5’-GGGGGTGTCTCAGTATCCCA-3’
Tgfβ2	Forward: 5’-GTTACAACACCCTCTGGCTCA-3’
	Reverse: 5’-TGTTCAGGCACTCTGGCTTT-3’
Tgfβ3	Forward: 5’-AAGAGGAGCGAAAGCACAGAG-3’
	Reverse: 5’-CAGCCCCAATCATCCACTCA-3’
