# Supplementary figures and images for: Regulating Blood Clot Fibrin Films to Manipulate Biomaterial-Mediated Foreign Body Responses
Source: Research (Wash D C). 2023 Sep 15;6:0225. doi: 10.34133/research.0225 (PMC10503960; doi:10.34133/research.0225)

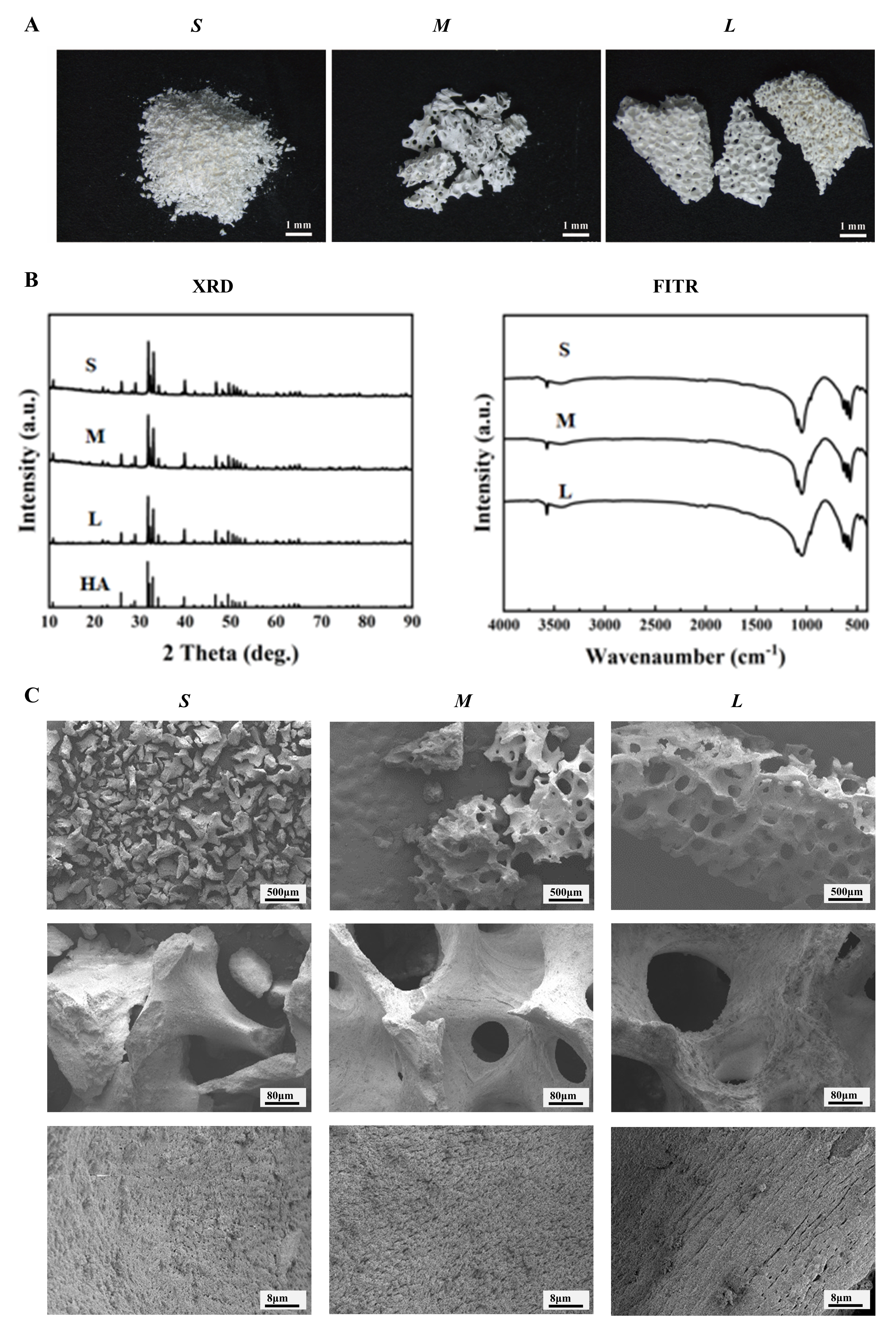

Supplement: Supplementary 1 — Figs. S1 to S5 Tables S1 and S2 [file research.0225.f1.zip › Figure S1.png]

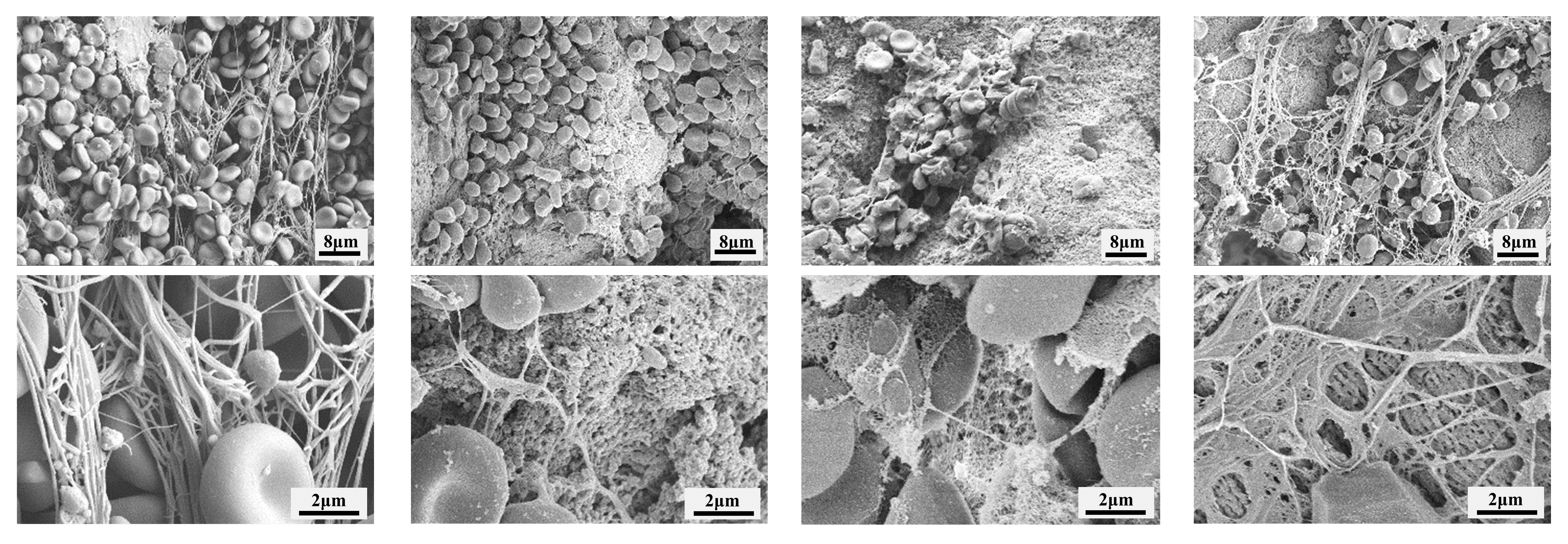

Supplement: Supplementary 1 — Figs. S1 to S5 Tables S1 and S2 [file research.0225.f1.zip › Figure S2.png]

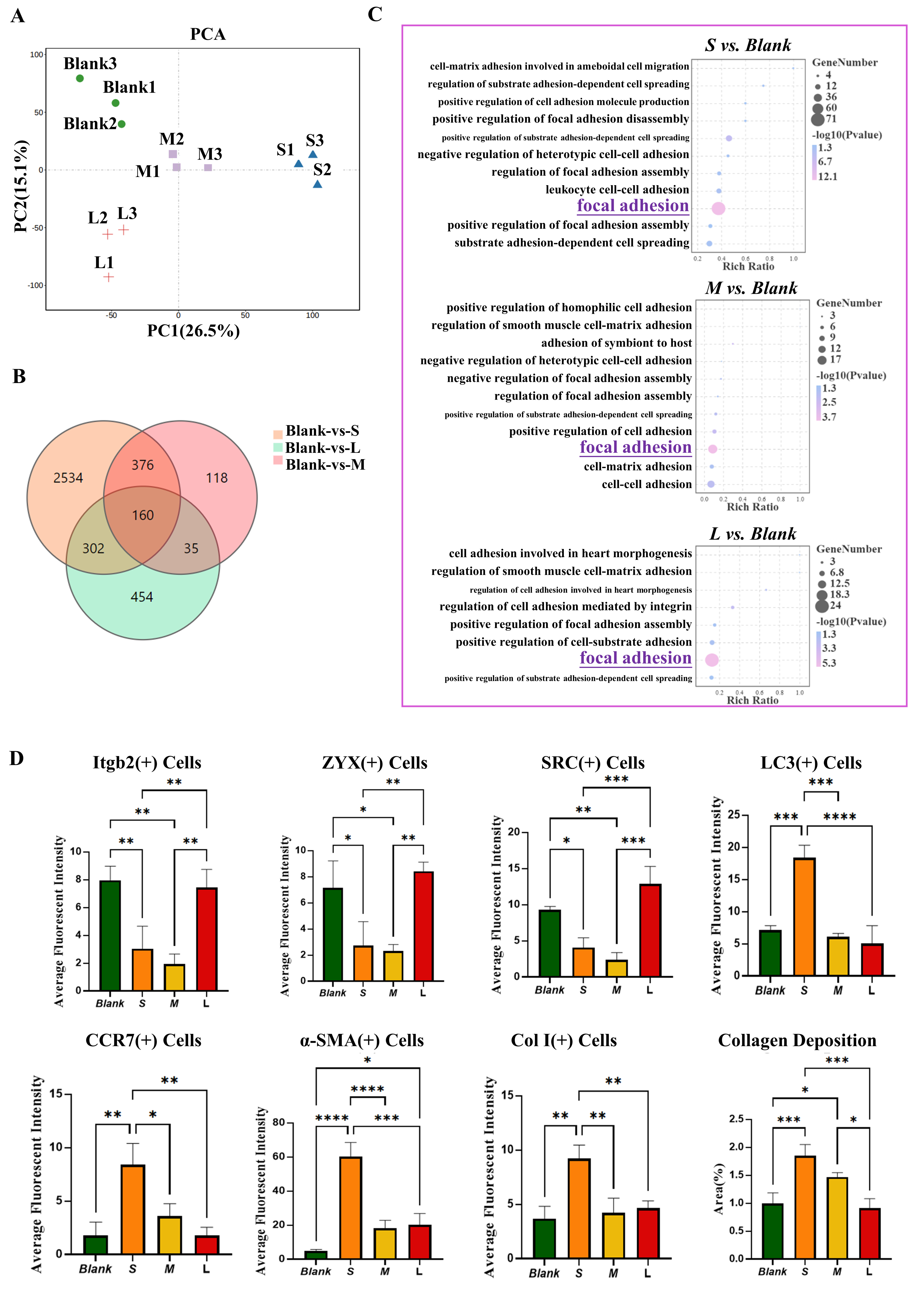

Supplement: Supplementary 1 — Figs. S1 to S5 Tables S1 and S2 [file research.0225.f1.zip › Figure S3.png]

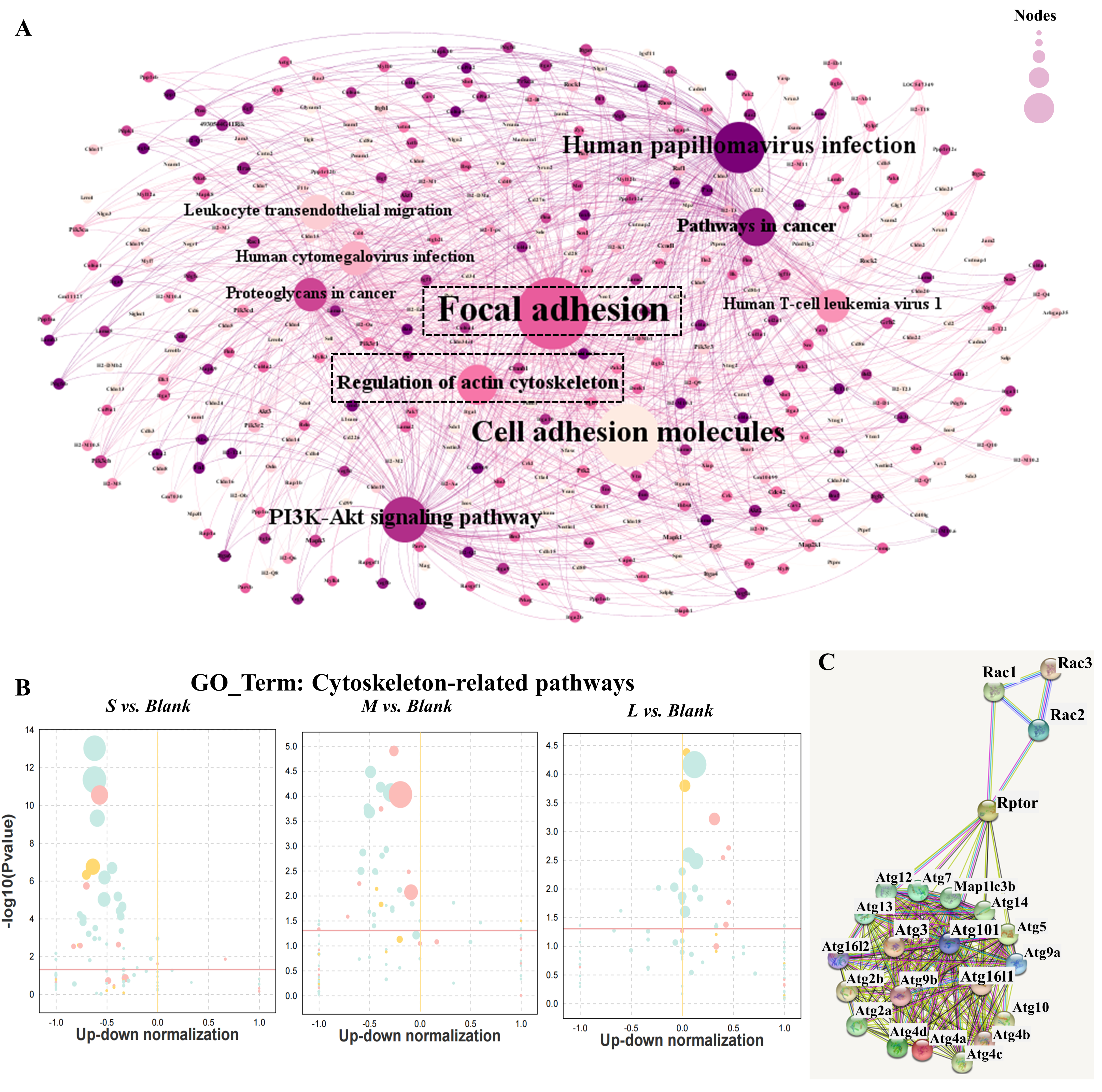

Supplement: Supplementary 1 — Figs. S1 to S5 Tables S1 and S2 [file research.0225.f1.zip › Figure S4.png]

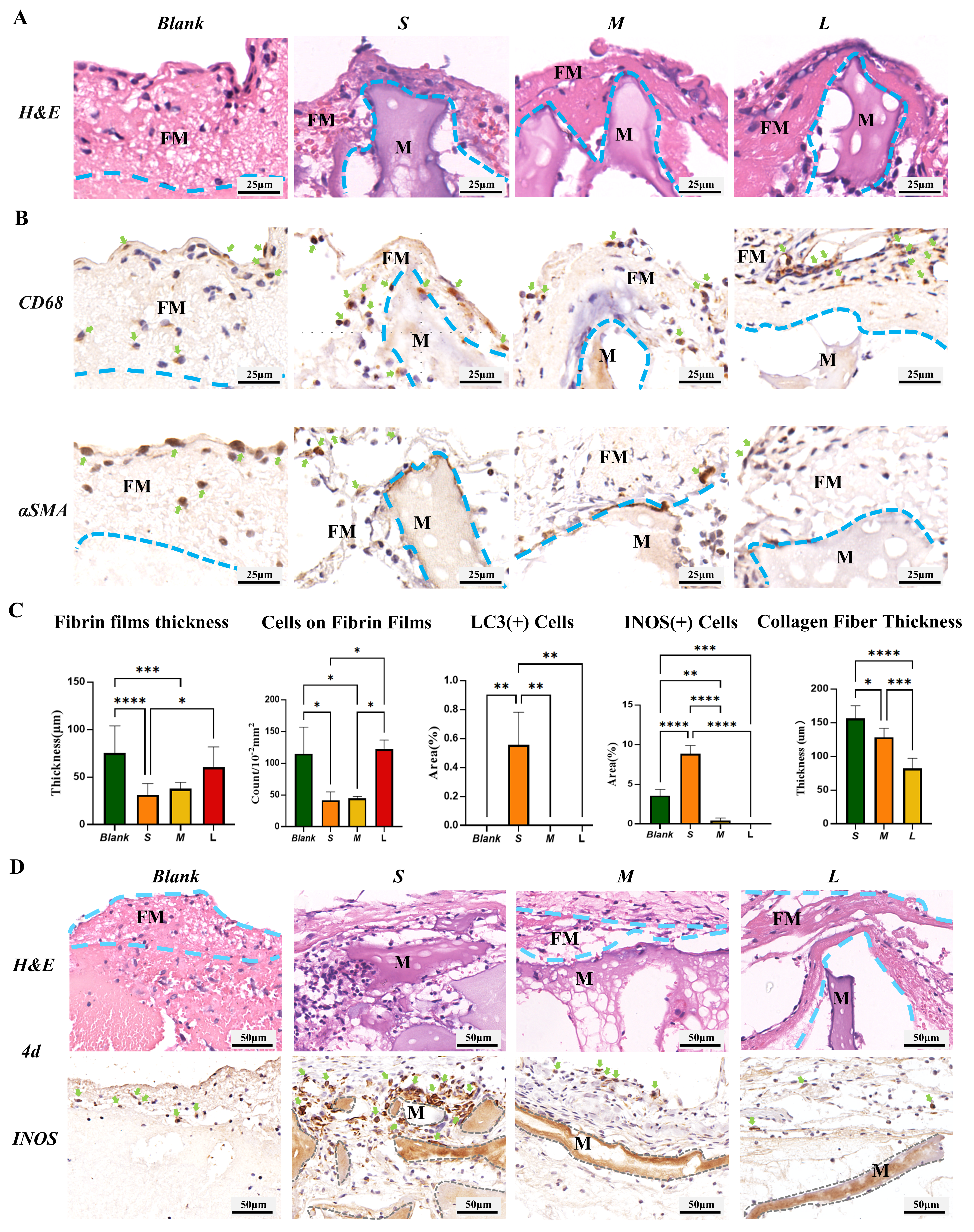

Supplement: Supplementary 1 — Figs. S1 to S5 Tables S1 and S2 [file research.0225.f1.zip › Figure S5.png]
